# RSRC2 Expression Inhibits Malignant Progression of Triple-Negative Breast Cancer by Transcriptionally Regulating SCIN Expression

**DOI:** 10.3390/cancers16010015

**Published:** 2023-12-19

**Authors:** Nan Zhao, Chunsheng Ni, Shuai Fan, Na Che, Yanlei Li, Song Wang, Yongli Li, Xueyi Dong, Yuhong Guo, Xiulan Zhao, Tieju Liu

**Affiliations:** 1 Department of Pathology, Tianjin Medical University, Tianjin 300070, China; 2 Department of Pathology, General Hospital, Tianjin Medical University, Tianjin 300052, China; 3 Department of Pathology, Tianjin Cancer Hospital, Tianjin Medical University, Tianjin 300060, China

**Keywords:** triple-negative breast cancer, RSRC2, SCIN, transcriptional regulation

## Abstract

**Simple Summary:**

Triple-negative breast cancer has poorer prognosis than other molecular subtype of breast cancer. RSRC2 is a newly discovered tumor suppressor gene. Our study found RSRC2 expression was lost in triple-negative breast cancer tissues. Low expression of RSRC2 was associated with worse prognosis of breast cancer patients. SCIN was identified as a novel transcriptional target of RSRC2 in triple-negative breast cancer cells. The clarification of relationship between RSRC2 and SCIN provided a new sight for triple-negative breast cancer treatment.

**Abstract:**

Triple-negative breast cancer (TNBC) has a shorter survival time and higher mortality rate than other molecular subtypes. RSRC2 is a newly discovered tumor suppressor gene. However, the potential functional mechanism of RSRC2 in TNBC remains unknown so far. Multiple bioinformatics databases were used. A Human Transcriptome Array 2.0 analysis, ChIP-seq analysis, ChIP-qPCR, RT-qPCR, Western blot, cell function assays in vitro and a metastatic mouse model in vivo were performed to demonstrate the role of RSRC2 in TNBC. Through the analysis of various databases, RSRC2 expression was the lowest in TNBC tissues compared to other molecular subtypes. The low expression of RSRC2 was associated with a worse prognosis for patients with breast cancer. The transcriptome array, ChIP-seq and bioinformatics analysis identified that GRHL2 and SCIN might have a close relationship with RSRC2. The functional bioinformatics enrichment analysis and functional cell experiments showed that RSRC2 was involved in cell adhesion, cell proliferation, cell migration and invasion. Furthermore, RSRC2 expression suppressed SCIN expression but not GRHL2 expression. SCIN re-expression in the RSRC2 overexpression cells or SCIN knockdown in the RSRC2 knockdown cells reversed the cellular function caused by RSRC2. Mechanistically, RSRC2 transcriptionally inhibited SCIN expression. In summary, our study reveals that RSRC2 acts as a tumor suppressor in TNBC development and progression through negatively regulating SCIN-mediated cell function, thus providing a potential target for TNBC treatment.

## 1. Introduction

Female breast cancer was the most commonly diagnosed cancer worldwide in 2020 [1]. The statistics from 11 regions of the world show that breast cancer is the most common cause of cancer death [2]. Triple-negative breast cancer (TNBC) was defined by the absence of ER/PR expression and human epidermal growth factor receptor 2 (Her2) amplification [3,4]. TNBC has a shorter survival time and higher mortality than non-TNBC. Due to its molecular characteristics, TNBC is not sensitive to endocrine therapy and Her2-targeted therapy. The main treatment for TNBC is chemotherapy, but the response to conventional chemotherapy is poor and drug resistance is common. Therefore, exploring new individualized therapeutic targets is of great significance for TNBC.

*RSRC2* (arginine/serine-rich coiled coil 2) was first reported by Kurehara H et al. in 2007 as a gene related to esophageal cancer but with an unknown function [5]. The *RSRC2* mRNA expression level of esophageal cancer cell lines and resected cancer tissues was significantly lower than that of normal esophageal mucosa [5]. *RSRC2* expression could inhibit esophageal cancer cell growth and is significantly negatively related to the esophageal cancer infiltration level, spreading to the lymph node and blood vessels [5]. Later research found that the expression level of RSRC2 in pancreatic ductal carcinoma was significantly lower than that in normal pancreatic tissue, and it was negatively correlated with the clinical stage of pancreatic ductal carcinoma [6]. The study of Park JS et al. [7] showed that the expression of *RSRC2* was related to the sensitivity of gastric cancer cells to the chemotherapeutic drug 5-fluorouracil. Compared with RSRC2 low-expression cell lines, *RSRC2* high-expression cell lines were more sensitive to 5-fluorouracil [7]. A study on colon cancer found that malignant biological behaviors such as the proliferation, migration and invasion of cancer cells were enhanced after SOCS3 downregulation, accompanied by a decrease in the RSRC2 expression level [8]. RSRC2 was upregulated in miR-182-silenced colorectal cancer cells, which induced apoptosis, inhibited cell proliferation and invasion and was not conducive to tumor formation in immunodeficient mice [9].

Our previous study [10] showed that the abnormal splicing of *RSRC2* induced by the splicing factor TRA2A led to the reduction in *RSRC2* short isoform mRNA expression, thus reducing the expression of the RSRC2 protein. The loss of RSRC2 protein expression plays a key role in the proliferation, invasion and paclitaxel resistance of TNBC cells.

As one of the members of the gelsolin superfamily, the major function of SCIN (scinderin) is to cleave and cap actin and then regulate the cytoskeleton and a series of biological functions. SCIN might play a dual function in tumors. It is reported that SCIN acts as a tumor suppressor in gastric cancer [11]. However, many studies report that SCIN promotes the malignant biological behavior of the tumor. The overexpression of SCIN can protect hepatocellular carcinoma cells from apoptosis and promote the growth of xenograft tumors in nude mice [12]. In prostate cancer cell lines, silencing the expression of SCIN can inhibit the proliferation and clonogenic ability of prostate cancer cells and promote the apoptosis of castration-resistant prostate cancer cells [13]. The downregulation of SCIN expression can make liver cancer cells sensitive to chemotherapy drugs, thus inhibiting tumor growth [12]. In the gastric cancer cell line SGC-7901, the knockdown of SCIN can inhibit the migration and invasion ability of tumor cells by inhibiting the epithelial–mesenchymal transition [14]. Furthermore, SCIN expression is observed to be higher in breast cancer tissues than in benign breast tissues. SCIN expression inhibition significantly leads to cell apoptosis and the reduced proliferation speed of the cells in breast cancer cell lines [15].

In this study, we tried to find the molecular mechanism for how RSRC2 functioned in TNBC. Meanwhile, the potential downstream targets of RSRC2 were explored, including SCIN, which were involved in the development and progression of multiple cancers [16,17,18]. Our results highlight an important role of RSRC2 in the malignant progression of TNBC, suggesting a novel therapeutic target for TNBC.

## 2. Materials and Methods

### 2.1. Bioinformatics Databases

The following databases were used: TIMER (http://cistrome.org/TIMER/ (accessed on 30 September 2021)), GEPIA (http://gepia.cancer-pku.cn/ (accessed on 30 September 2021)), HPA (https://www.proteinatlas.org/ (accessed on 29 August 2005)), UALCAN (http://ualcan.path.uab.edu/ (accessed on August 2017)), TCGA (https://www.cancer.gov/tcga (accessed on 29 May 2006)), Kaplan–Meier Plotter (https://kmplot.com/analysis/ (accessed on 1 January 2009)), PrognoScan (http://dna00.bio.kyutech.ac.jp/PrognoScan/index.html (accessed on 1 January 2009)), LinkedOmics (http://www.linkedomics.org/ (accessed on 29 March 2017)), Metascape (http://metascape.org/ (accessed on 8 October 2015)), Breast Cancer Gene-Expression Miner v4.8 (http://bcgenex.ico.unicancer.fr/ (accessed on 5 February 2010)), STRING (https://string-db.org/cgi/input.pl (accessed on 25 September 2003)), GeneMANIA (http://www.genemania.org (accessed on 11 August 2010)) and Xiantao Academic (https://www.xiantao.love/products (accessed on 23 April 2014)).

### 2.2. Cell Culture and Lentiviral Transduction

MDA-MB-231, MCF-7, T47D and MDA-MB-453 were obtained from the American Type Culture Collection. The RSRC2 overexpression plasmid (Catalog No.: EX-H8692-Lv201), RSRC2 single-guide RNA (sgRNA) plasmid (Catalog No.: HCP309753-LvSG06-3-10) and RSRC2 short hairpin (sh) plasmid (Catalog No.: HSH017340-LVRU6GP) were purchased from FulenGen (Guangzhou, China). The pEZ-Lv201 vector was used for overexpressing RSRC2, the psi-LVRU6GP was used for RSRC2 silencing and the pCRISPR-LvSG06 was used for RSRC2 knockout. To generate lentiviral shRNA constructs targeting human SCIN, the target sequences were designed according to the literature [16] and cloned into the psi-LVRU6MP vector. SCIN overexpression constructs were generated by cloning SCIN sequences into the Lv201 vector.

### 2.3. Wound Healing Assay, Cell Migration and Invasion Assays and Plate Clonogenic Assay

The assays were carried out according to our previous study [10].

### 2.4. Adhesion Assay

The six-well plates were placed in a 37 °C incubator for 15 min after coating with 1 μg/mL fibronectin. The 1 × 10^6^ cells in normal medium were seeded into plates and incubated at 37 °C for 50 min. Then, the non-adherent cells in the six-well plates were washed away by using PBS. The remaining cells were stained with 0.1% crystal violet for 5 min. The cells were observed and analyzed in three randomly selected fields.

### 2.5. Luciferase Reporter Gene Experiment

The SCIN promoter plasmid (Catalog No.: HPRM51588-PG04) and SCIN negative control plasmid (Catalog No.: NEG-PG04) were purchased from FulenGen (China). The above plasmids and RSRC2 overexpression plasmid (0.6 μg, 1 μg and 1.4 μg was used, respectively) or control plasmid were cotransfected into the 293T cells. After 48 h, the collected medium was added to the GLuc and SEAP reaction solution and measured with the luminometer.

### 2.6. RNA Extraction, Microarray Analysis and Quantitative Real-Time Polymerase Chain Reaction (qRT-PCR)

The Affymetrix GeneChip^®^ Human Transcriptome Array 2.0 (Oebiotech, Shanghai, China) was used for the transcriptome analysis. A QRT-PCR was performed according to our previous study [10], using All-in-One qPCR Primer (FulenGen, China).

### 2.7. Chromatin Immunoprecipitation (ChIP) Assays

The MDA-MB-231 cells were sent to Sangon Biotech (Shanghai, China) for the ChIP sequencing analysis, and RSRC2 antibody (Santa Cruz, TX, USA) was used. The ChIP-qPCR assay was performed as previously described [19]. The antibodies against RSRC2 (Santa Cruz, TX, USA), GRHL2 (Sigma Aldrich, St Louis, MO, USA) and IgG (Santa Cruz, TX, USA) were used. The primer sequences for the ChIP-qPCR are listed in Table S1.

### 2.8. Western Blot Analysis

The assay was performed according to our previous study [10]. The primary antibodies RSRC2 (1:500, Santa Cruz, TX, USA), GRHL2 (1:500, Sigma) and SCIN (1:500, Santa Cruz) and the secondary antibody (1:2000; Santa Cruz, TX, USA) were used. For the protein-loading analyses, the GAPDH antibody (1:2000; Santa Cruz, TX, USA) was used.

### 2.9. Animal Experiment

The animal experiment was approved by Tianjin Medical University and conducted according to the Animal Study Guidelines of Tianjin Medical University. Five-week-old female nude mice (BALB/c) were used for the animal study. The MDA-MB-231 sgRSRC2 cells and control cells (2 × 10^6^) suspended in 200 μL of PBS were injected into the tail vein of the nude mice with five mice per group. After 4 weeks, the mice were sacrificed and the lung, liver, spleen and kidney samples were harvested, fixed in 10% formalin, dehydrated and embedded in paraffin. The tissue sections (5 μm in thickness) were prepared according to the standard protocols for hematoxylin/eosin (H&E) staining.

### 2.10. Statistical Analysis

We performed the statistical analysis by using SPSS 16.0 software. *p* < 0.05 was deemed as statistical significance.

## 3. Results

### 3.1. Expression of RSRC2 in Cancers by Bioinformatics Analysis

**Figure 1:**
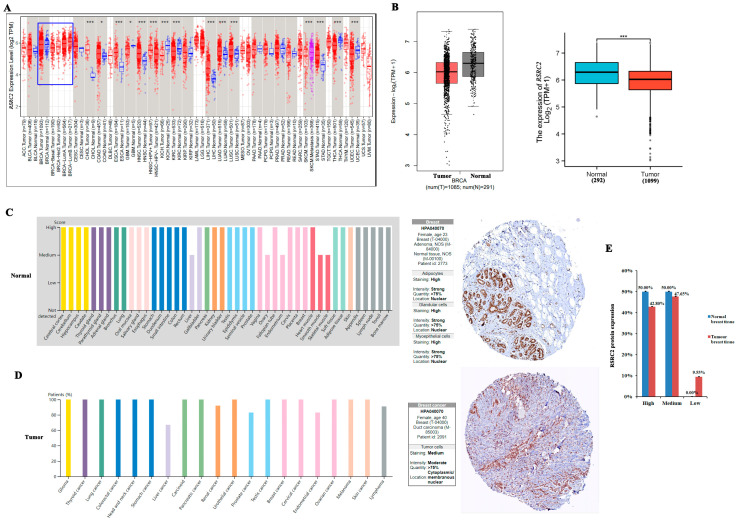
Expression of RSRC2 by bioinformatics analysis. (**A**) The expression of *RSRC2* mRNA in multiple cancers. Blue box indicates *RSRC2* expression was lower in basal (n = 190) and Her2 (n = 82) subtypes than in luminal A and B (n = 564 and n = 217) and normal tissue (n = 112). (**B**) GEPIA database and Xiantao Academic online analysis database showed that *RSRC2* expression was significantly lower in breast cancer tissues than in normal tissues. (**C**,**D**) Immunohistochemical staining obtained from HPA showed strong expression of RSRC2 protein in normal tissues (**C**) and moderate expression of RSRC2 protein in tumor tissues (**D**). The black underlines indicate immunohistochemical staining intensity. The *x* axis in (**C**) shows different human organs and tissues, and the *y* axis shows RSRC2 expression score. The *x* axis in (**D**) shows the existing tumor types in the HPA database, and the *y* axis shows the percentage of patients with high/medium RSRC2 expression in the total tested patients. (**E**) The decreased RSRC2 protein expression occurred in breast cancer tissues compared to normal breast tissues. (* *p* < 0.05, *** *p* < 0.001).

We analyzed the expression level of the *RSRC2* mRNA in the TCGA database for multiple cancer types. The results showed that the expression of *RSRC2* in cholangiocarcinoma (CHOL), colon adenocarcinoma (COAD), esophageal cancer (ESCA), head and neck squamous cell carcinoma (HNSC), liver hepatocellular carcinoma (LIHC), lung adenocarcinoma (LUAD), lung squamous cell carcinoma (LUSC) and stomach adenocarcinoma (STAD) was significantly higher than that in the corresponding normal tissues (Figure 1A). On the other hand, the expression level of *RSRC2* in glioblastoma (GBM), kidney chromophobe cell carcinoma (KICH), kidney renal clear-cell carcinoma (KIRC), thyroid carcinoma (THCA) and uterus endometrial carcinoma (UCEC) was lower than that in the normal tissues (Figure 1A). Although there was no difference between the breast cancer (BRCA, n = 1093) and normal breast tissue (n = 112), the *RSRC2* expression was lower in the basal (n = 190) and Her2 (n = 82) subtypes than in the luminal A and B (n = 564 and n = 217) and normal tissue (Figure 1A, blue box indicated).

It was also confirmed from the GEPIA database and Xiantao Academic online analysis database that the *RSRC2* expression was significantly lower in the breast cancer tissues (n = 1085 and n = 1099) than in the normal tissues (n = 291 and n = 292) (Figure 1B). The immunohistochemical staining obtained from HPA showed the strong expression of the RSRC2 protein in the normal tissues (Figure 1C) and the moderate expression of the RSRC2 protein in the tumor tissues (Figure 1D). In the normal breast tissues, no RSRC2 low expression was found, and all the normal tissues had high (50%) and medium (50%) expression of the RSRC2 protein, while in the breast cancer tissues, the RSRC2 low expression accounted for 9.55%, medium expression accounted for 47.65% and high expression accounted for 42.8% (Figure 1E). Therefore, the decreased RSRC2 protein expression occurred in the breast cancer tissues compared to the normal breast tissues.

### 3.2. RSRC2 Expression Was Especially Lower in TNBC Than in Non-TNBC

**Figure 2:**
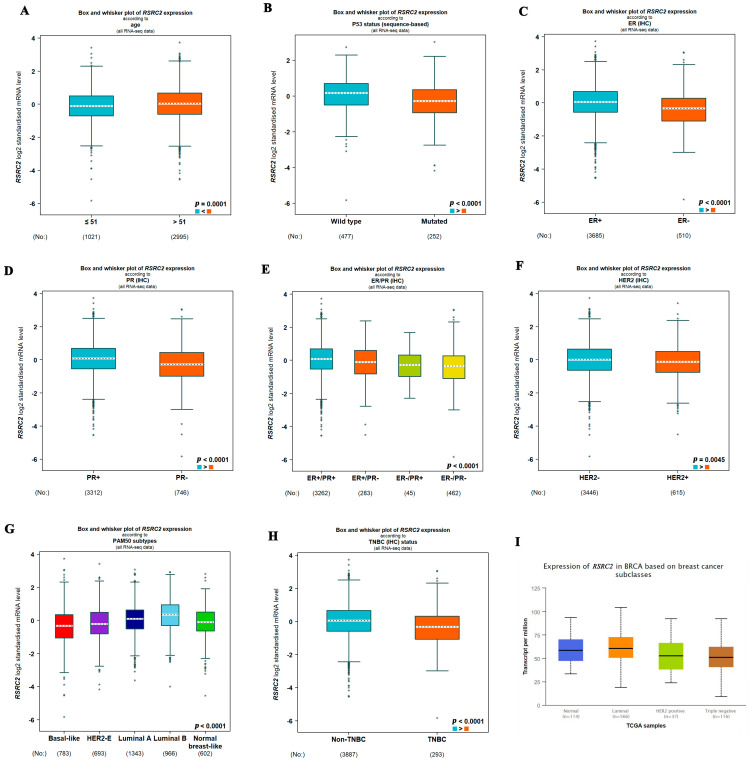
*RSRC2* expression was lower in TNBC than in other subtypes. *RSRC2* expression was lower in age ≤51 (**A**), mutated p53 status (**B**), ER (**C**), PR (**D**) or ER/PR negative (**E**) than in age > 51, wild-type p53 status, ER, PR or ER/PR positive. (**F**) *RSRC2* expression was lower in Her2 (+) than in Her2 (−). (**G**–**I**) *RSRC2* expression was lowest in TNBC among all the subtypes of breast cancer.

Next, we further demonstrated the correlation between the *RSRC2* mRNA levels and the clinical data of breast cancer patients. The expression of *RSRC2* was not correlated with the T stage, N stage, M stage, pathologic stage and histological type (*p* > 0.05) (Table S2). Interestingly, the *RSRC2* mRNA levels were significantly correlated with age, p53 status, ER status, PR status and Her2 status (Table S2 and Figure 2A–F) (*p* < 0.05). The *RSRC2* expression was lower in age ≤ 51 (Figure 2A), mutated p53 status (Figure 2B), ER (Figure 2C), PR (Figure 2D) or ER/PR negative (Figure 2E) than in age > 51, wild-type p53 status, ER, PR or ER/PR positive (*p* < 0.05). The *RSRC2* expression was also lower in Her2 (+) (Figure 2F) than in Her2 (−) (*p* < 0.05). Importantly, the *RSRC2* expression was lowest in TNBC among all the subtypes of breast cancer (*p* < 0.001) (Figure 2G–I).

### 3.3. RSRC2 Expression Has Prognostic Value in Breast Cancer

**Figure 3:**
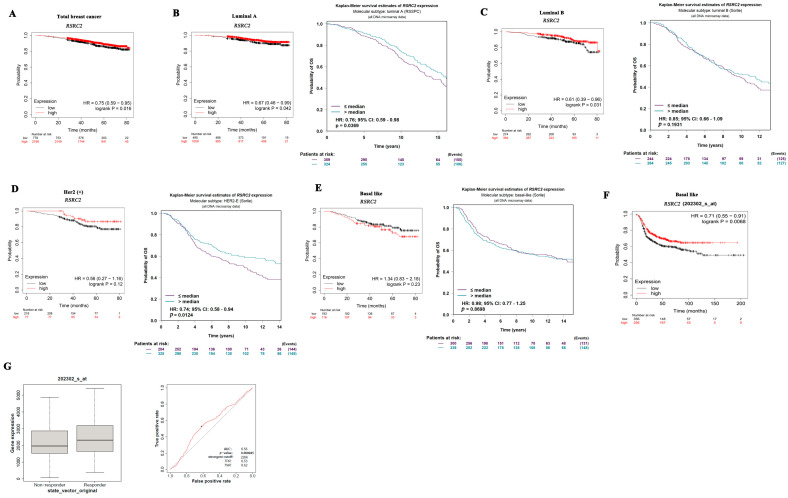
*RSRC2* expression has prognostic value in breast cancer. (**A**) *RSRC2* low expression can predict poorer overall survival (OS) in total breast cancer. (**B**) *RSRC2* low expression was related to poorer OS in luminal A. (**C**–**F**) *RSRC2* expression showed different prognostic values in different databases for luminal B (**C**), Her2 (+) (**D**) and basal-like subtype (**E**,**F**). (**G**) RSRC2 low expression was prone to drug resistance than RSRC2 high expression. Red line and black line show the effectiveness of RSRC2 evaluating chemotherapy responder. Black line represents a curve, with an area under the curve (AUC) value of 0.5, which means chemotherapy non-responder. The AUC value under red line is 0.56, indicating a reliable predictive value of RSRC2 as breast cancer chemotherapy responder.

We used multiple databases to assess the prognostic value of *RSRC2*. *RSRC2* low expression can predict poorer overall survival (OS) in total breast cancer (Figure 3A). The *RSRC2* expression showed different prognostic values in different databases for luminal A (Figure 3B), luminal B (Figure 3C), Her2 (+) (Figure 3D) and TNBC (Figure 3E,F). Importantly, the analysis of another database with larger TNBC samples showed that *RSRC2* low expression was significantly related to a poorer prognosis in TNBC (Figure 3F). Meanwhile, we found that the *RSRC2* expression was lower in the chemotherapy non-responder than in the chemotherapy responder (Figure 3G) by using the Kaplan–Meier Plotter, suggesting that breast cancer with *RSRC2* low expression was prone to drug resistance than *RSRC2* high expression. The area under the ROC curve was 0.56, indicating a reliable predictive value of *RSRC2* as a breast cancer chemotherapy responder (Figure 3G).

### 3.4. Functional Enrichment Analysis of RSRC2 Coexpressed Genes in TNBC

To clarify the genes and signal transduction pathways related to *RSRC2* in TNBC, we used the LinkedOmic database and Metascape software (version v3.5) to analyze the negatively and positively coexpressed genes with *RSRC2* in TNBC. The functional enrichment analysis showed that genes negatively related to RSRC2 were involved in multiple cellular functions, in which the regulation of the cell migration, cell adhesion, focal adhesion, cell adhesion molecule binding and cell-substrate adherens junction appeared more frequently (Figure S1). However, the cell cycle and DNA replication process were the main cellular functions in the genes positively related to RSRC2, while the focal adhesion and cell adhesion molecules accounted for only a small fraction of the functions (Figure S2).

### 3.5. RSRC2-Downregulated Expression Promotes Migration, Invasion and Metastasis of MDA-MB-231 Cells

**Figure 4:**
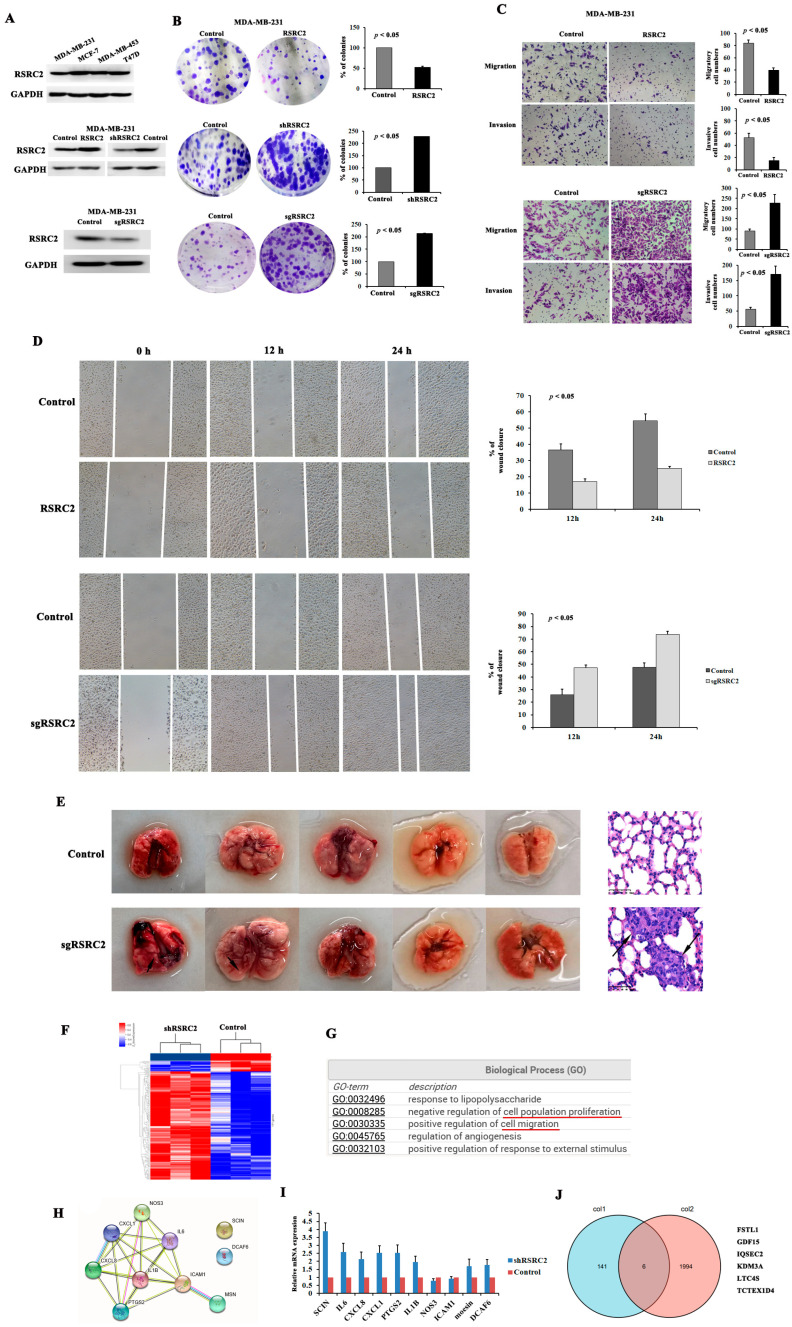
RSRC2-downregulated expression promotes migration, invasion and metastasis of MDA-MB-231 cells and global regulation of the transcriptome by RSRC2 in MDA-MB-231 cells. (**A**) MDA-MB-231 cells showed a slight decline in RSRC2 expression than MDA-MB-453, MCF-7 and T47D cells; the protein expression of RSRC2 in MDA-MB-231 cells overexpressing RSRC2 was significantly higher than that in the control cells, and the protein expression of RSRC2 in shRSRC2 MDA-MB-231 cells was significantly lower than that in the empty vector-transfected control cells. (**B**) Plate clone formation experiment results showed that the number of clones in shRSRC2 or sgRSRC2 cells was higher than that of the control cells, while the number of clones in RSRC2 overexpression cells was lower than that of the control cells. Error bars represent SD. (**C**) Migration and invasion assays showed that RSRC2 knockout significantly increased vertical migration and invasion, while RSRC2 overexpression reduced vertical migration and invasion of MDA-MB-231 cells. Error bars represent SD. (**D**) Wound healing assay showed that RSRC2 knockout could promote horizontal migration of MDA-MB-231 cells and RSRC2 overexpression could inhibit horizontal migration of MDA-MB-231 cells. Error bars represent SD. (**E**) Two mice showed lung metastases nodules in sgRSRC2 group (black arrow) and were confirmed by histological examination. (**F**) A total of 171 genes were differently expressed, including 156 upregulated genes and 15 downregulated genes in shRSRC2 MDA-MB-231 cells and control cells. (**G**) Gene Ontology (GO) analysis was performed and identified that cell proliferation and cell migration (indicated by red line) might be related to RSRC2 expression of MDA-MB-231 cells. (**H**) Most of the genes involved in cell proliferation and cell migration identified by GO analysis could be functionally connected into well-linked interaction network by STRING analysis, except SCIN and DCAF6. (**I**) QRT-PCR results demonstrated that SCIN showed the highest expression level than the other 9 genes in MDA-MB-231 shRSRC2 cells when compared with the control cells. Error bars represent SD. (**J**) FSTL1, GDF15, IQSEC2, KDM3A, LTC4S and TCTEX1D4 were identified by intersection set analysis of HTA 2.0 microarray data of MDA-MB-231 and bioinformatics database of TNBC. The graphs represent three repeated experiments. Student’s *t* tests were used to compare two groups’ means. The uncropped blots are shown in Figure S4.

We detected RSRC2 expression in the TNBC cell lines MDA-MB-231 and MDA-MB-453, non-TNBC cell line MCF-7 and T47D cells by Western blotting (Figure 4A). The MDA-MB-231 cells as the most aggressive cell line showed a slight decline in RSRC2 expression than the other three cell lines.

Next, the MDA-MB-231 and MDA-MB-453 cells were transfected with the RSRC2 overexpression plasmid and shRNA knockdown plasmid or sgRNA knockout plasmid (Figure 4A and Figure S3A). The plate clone formation experiment results showed that the cells with RSRC2 overexpression, RSRC2 knockdown or RSRC2 knockout and control cells could form clones, but the number of clones in the shRSRC2 cells or sgRSRC2 cells was higher than that of the control cells, while the number of clones in the RSRC2 overexpression cells was lower than that of the control cells. These differences were statistically significant in both the MDA-MB-231 cells (Figure 4B) and MDA-MB-453 cells (Figure S3B). Meanwhile, the migration and invasion assays showed that RSRC2 knockout significantly increased the vertical migration and invasion, while RSRC2 overexpression reduced the vertical migration and invasion of the MDA-MB-231 (Figure 4C) and MDA-MB-453 cells (Figure S3C,D) when compared with the control cells. The wound healing assay showed that the horizontal migration of the MDA-MB-231 (Figure 4D) and MDA-MB-453 cells (Figure S3E) could be promoted by RSRC2 knockout or inhibited by RSRC2 overexpression.

Then, the in vivo metastatic mouse model by tail vein injection of the MDA-MB-231 sgRSRC2 cells and control cells was established to evaluate the tumor metastasis. The histological examination showed no tumor involvement in the liver, spleen and kidney samples. However, we found a tumor metastasis nodule in the lungs (Figure 4E). There were two mice that showed lung metastases in the sgRSRC2 group (Figure 4E, black arrow), while no lung metastases were observed in the control group.

### 3.6. Global Regulation of the Transcriptome by RSRC2 in MDA-MB-231 Cells

In order to identify the genes related to RSRC2 and that more specifically functioned in TNBC cells, we assessed the mRNA expression profiles of the MDA-MB-231 shRSRC2 cells and the control cells by using the Human Transcriptome Array (HTA) 2.0. The comparison results showed that 171 genes were differently expressed, including 156 upregulated genes and 15 downregulated genes (Table S3 and Figure 4F). Among the GO enrichment results produced by all the differently expressed genes between the shRSRC2 cells and control cells, we found several GO terms that may relate to our research, such as the regulation of cell population proliferation and cell migration (Figure 4G).

Further, we found 10 genes (SCIN, IL6, CXCL8, CXCL1, PTGS2, IL1B, NOS3, ICAM1, moesin and DCAF6) that were involved in cell proliferation, migration and cell adhesion through a GO analysis. Using the Search Tool for the Retrieval of Interacting Genes/Proteins (STRING)–Known and Predicted Protein–Protein Interactions analysis, we found that except for SCIN and DCAF6, the left genes could be functionally connected into a well-linked interaction network (Figure 4H). These results suggested that SCIN and DCAF6 may play a relatively independent role in the signal pathway regulated by RSRC2. The QRT-PCR was performed and SCIN showed the highest expression level rather than the other nine genes in the MDA-MB-231 shRSRC2 cells when compared with the control cells (Figure 4I).

Next, the genes obtained from the HTA 2.0 microarray data of MDA-MB-231 and the bioinformatics database of TNBC were analyzed through the intersection set analysis, and six common genes were identified. These genes were FSTL1, GDF15, IQSEC2, KDM3A, LTC4S and TCTEX1D4 (Figure 4J), in which most of them were involved in tumor cell proliferation and growth.

### 3.7. The Genomic Occupancy and Motif Combination by RSRC2

To reveal the genomic binding sites of RSRC2, ChIP-seq was performed in MDA-MB-231 cells under a normal culture condition. The ChIP-seq data were compared with the reference genome (hg38) and the enriched regions were scanned from the short sequences matched to the genome. The enriched regions were considered to be the binding regions of the RSRC2 protein and DNA. A peak analysis was conducted by using the Macs2 tool. A total of 446 peaks were mapped (Table S4). Please refer to http://homer.ucsd.edu/homer/ngs/annotation.html (accessed on 19 February 2021) for the specific annotation information. We identified that the DNA region bound to the RSRC2 protein could be at the intergenic, intron, promoter-TSS, 3′ UTR and exon regions (Table S3).

**Figure 5:**
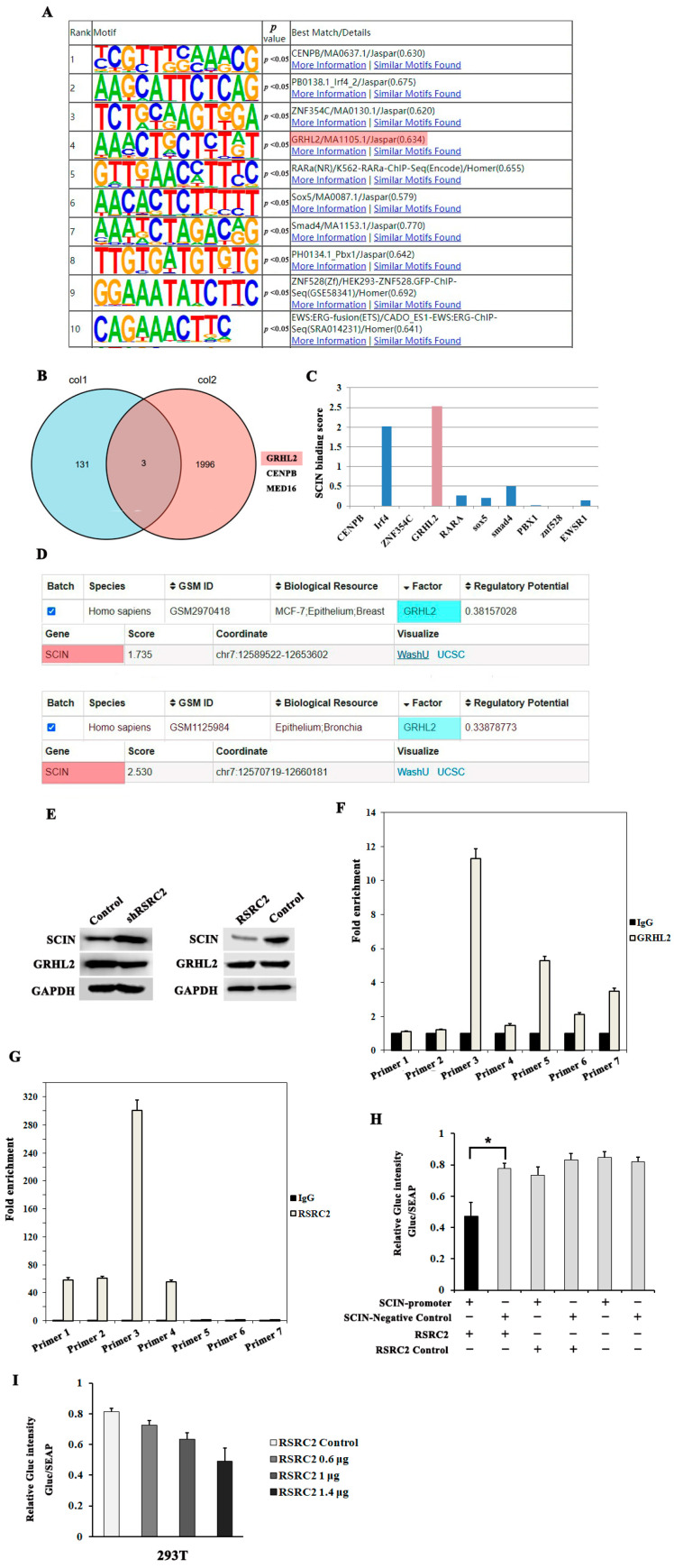
RSRC2 might directly regulate SCIN expression. (**A**) The top 10 motifs that could be bound by RSRC2 protein. (**B**) Intersection set analysis of Homer ChIP-seq data and LinkedOmic database and Metascape software (version v3.5) with TNBC data identified GRHL2, CENPB and MED16 as common genes. (**C**) GRHL2 has the greatest potential for SCIN binding, while the other factors did not obtain the regulatory score or showed a lower regulatory score. (**D**) GRHL2 protein had regulatory potential for SCIN in breast cancer cells. (**E**) GRHL2 protein expression did not show significant alteration, although SCIN protein expression decreased after RSRC2 upregulation and increased after RSRC2 downregulation. (**F**) ChIP qRT-PCR showed GRHL2 could bind to the promoter of SCIN. (**G**) ChIP qRT-PCR showed RSRC2 could bind to the promoter of SCIN. (**H**) The luciferase activity of RSRC2 overexpression and SCIN promoter cotransfection group significantly decreased compared with other groups. (**I**) The luciferase activity was decreased in an RSRC2 overexpression plasmid dose-dependent manner. Error bars represent SD and asterisks denote statistical significance <0.05.

The Homer ChIP-seq analysis identified the motifs that could be bound by the RSRC2 protein, and the top 10 motifs are shown in Figure 5A. These motifs could also be bound by transcription factors CENPB, IRF4, ZNF354C, GRHL2, RARa, SOX5, SMAD4, PBX1, ZNF528 and EWSR1 (Figure 5A). Further, the Homer ChIP-seq data and LinkedOmic database and Metascape software (version v3.5) with TNBC data were analyzed through an intersection set analysis, and three common genes (GRHL2, CENPB and MYB) were identified (Figure 5B), which suggested that these transcription factors might function in TNBC. RSRC2 and these transcription factors shared common target sequences. Therefore, RSRC2 might play the role of transcriptional regulation in TNBC.

To further correlate motif bindings with direct gene regulation, we integrated Homer ChIP-Seq data with HTA 2.0 microarray data and noticed that SCIN might hold a motif that could be combined by GHRL2. The Cistrome DB analysis was used to analyze the potential factors that might directly regulate SCIN through a motif analysis and found GRHL2 has the greatest potential for SCIN binding (Figure 5C), while the other factors did not obtain the regulatory score or showed a lower regulatory score (Figure 5C). Further, the Cistrome DB analysis showed that the GRHL2 protein had regulatory potential for SCIN in breast cancer cells (Figure 5D). In the GSM2970418 data including the breast cancer cell and epithelium, the regulatory score was 1.735 of GRHL2 for SCIN (Figure 5D). In the GSM1125984 data including the epithelium and bronchia, GRHL2 showed a slightly lower regulatory potential for SCIN but with the higher regulatory score (Figure 5D).

In order to demonstrate whether or not RSRC2 regulated the expression of SCIN by regulating GRHL2 expression, we observed GRHL2 and SCIN expression after RSRC2 expression upregulated or downregulated. The Western blotting showed that the GRHL2 protein expression did not show a significant alteration, although the SCIN protein expression decreased after RSRC2 upregulation and increased after RSRC2 downregulation (Figure 5E).

### 3.8. RSRC2 Might Directly Regulate SCIN Expression

To determine whether RSRC2 and GRHL2 bind to the promoter of the SCIN gene, a ChIP qRT-PCR was performed in MDA-MB-231 cells. The RSRC2 and GRHL2-binding peak sequence of the promoter region of SCIN was precipitated with RSRC2 or GRHL2 antibody, and seven primers were designed. As expected, GRHL2 bound to the promoter of SCIN (Figure 5F). Importantly, the results showed that RSRC2 could directly bind to the promoter region of SCIN (Figure 5G).

Then, we constructed the SCIN promoter plasmid and observed whether RSRC2 expression affected the SCIN promoter activity through a dual-luciferase reporter gene experiment. The results showed that after cotransfection of the RSRC2 empty vector (control) or RSRC2 overexpression plasmid (RSRC2) and SCIN promoter plasmid or SCIN negative control plasmid into the 293T cells, the luciferase activity of the RSRC2 overexpression and SCIN promoter cotransfection group significantly decreased compared with the other groups (Figure 5H). Meanwhile, the luciferase activity was decreased in an RSRC2 overexpression plasmid dose-dependent manner (Figure 5I). The experiment demonstrated that RSRC2 could bind to the SCIN promoter.

### 3.9. RSRC2 Inhibited Cell Adhesion, Clonality, Migration and Invasion Abilities by Suppressing SCIN Expression

**Figure 6:**
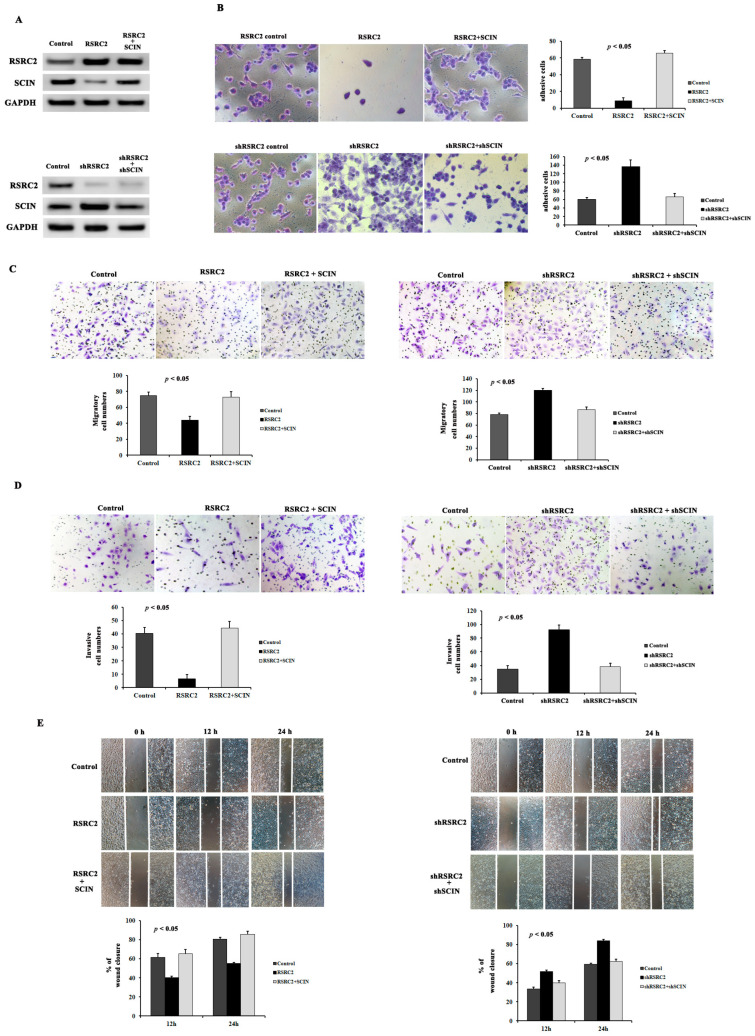
RSRC2 inhibited cell adhesion, migration and invasion abilities by suppressing SCIN expression. (**A**) Western blot showed SCIN re-expression in RSRC2 overexpression cells and SCIN expression inhibition in RSRC2 knockdown cells by rescue experiments. (**B**–**E**) The adhesive ability (**B**), cell migration (**C**) and invasion ability (**D**) and wound-healing ability (**E**) decreased or increased significantly after RSRC2 expression increased or decreased. SCIN upregulation in the RSRC2-overexpressing cells or downregulation in shRSRC2 cells, respectively, increased or decreased cell adhesive ability (**B**), cell migration (**C**) and invasion ability (**D**) and wound-healing ability (**E**). Error bars represent SD. The graphs represent three repeated experiments. ANOVA was used to compare multiple groups’ means. *p* < 0.05 was considered to be significant.

We next investigated whether the RSRC2 expression in the MDA-MB-231 cells might affect the ability of the MDA-MB-231 cells to adhere to extracellular matrix proteins. The results showed that the adhesive ability was increased significantly after the RSRC2 expression decreased and the SCIN expression increased (Figure 6A,B). Meanwhile, the RSRC2 overexpression inhibited the SCIN expression in the MDA-MB-231 cells and suppressed the cell adhesive ability accordingly (Figure 6A,B).

The Transwell migration and invasion assay showed that the amount of cell migration and invasion in the MDA-MB-231 (Figure 6C,D) or MDA-MB-453 (Figure S3C,D) cells overexpressing RSRC2 decreased compared with the control cells, and the decreased RSRC2 expression of the MDA-MB-231 or MDA-MB-453 cells significantly increased the ability of cell migration and invasion. Moreover, the quantitative analyses of the wound healing assay suggested a significant difference in the speed of wound healing between the RSRC2 overexpression cells and the control cells, the shRSRC2 or sgRSRC2 cells and the control cells. The RSRC2 overexpression cells displayed the slower speed while the shRSRC2 or sg RSRC2 cells showed the faster speed of wound healing than the control cells (Figure 6E and Figure S3E).

Next, we further demonstrated the relationship between RSRC2 and SCIN through the rescue experiments. We found that the SCIN upregulation in the RSRC2-overexpressing cells (Figure 6A and Figure S3A) or the downregulation in the shRSRC2 (Figure 6A) or sgRSRC2 (Figure S3A) cells, respectively, increased or decreased the cell adhesive ability (Figure 6B), clonality (Figure S3B) and cell migration and invasion ability (Figure 6C,D and Figure S3C,D). In addition, the SCIN overexpression plasmid transfected in the MDA-MB-231 cells or MDA-MB-453 cells with RSRC2 overexpression or SCIN knockdown in the MDA-MB-231 or MDA-MB-453 cells with RSRC2 downregulation reduced or increased the wound healing time (Figure 6E and Figure S3E).

## 4. Conclusions

TNBC is a subset of breast cancer with an adverse prognosis and significant tumor heterogeneity [20]. RSRC2 is a newly discovered tumor suppressor gene [5,10]. It is expressed in a variety of normal tissues, but its expression is decreased in tumor tissues. However, so far, there are few studies on the target genes and signaling pathways regulated by RSRC2.

Our previous study [10] showed that the expression of RSRC2 was significantly lower in 47 cases of the TNBC specimen than in the adjacent tissues. The expression of the RSRC2 protein was a protective factor, and the survival analysis showed that the decreased RSRC2 protein expression was significantly associated with poor survival in TNBC. In this study, we used bioinformatics databases to further carry out a large-sample analysis for the role of RSRC2 in cancer. We found the *RSRC2* expression was lower in multiple cancer tissues than in normal tissues. Meanwhile, the *RSRC2* expression was significantly lower in breast cancer tissues than in normal tissues. *RSRC2* low expression can predict poorer overall survival in breast cancer, even in different molecular subtypes, including TNBC. In addition, the *RSRC2* mRNA levels were significantly correlated with age, p53 status, ER status, PR status and Her2 status. The *RSRC2* expression was lower in the basal and Her2 subtypes than in the luminal A, B and normal tissue. Importantly, the *RSRC2* expression was lowest in TNBC compared with the other subtypes. These results suggested the specific role of RSRC2 in TNBC.

Our previous research work found that the number of proliferating clones and the cell survival of MDA-MB-231 cells with RSRC2 knockdown were significantly increased after paclitaxel treatment [10], demonstrating RSRC2 functioning in paclitaxel resistance. Consistently, the bioinformatics analysis showed breast cancer with *RSRC2* low expression was prone to drug resistance than with *RSRC2* high expression.

The bioinformatics functional enrichment analysis showed that genes related to RSRC2 were involved in multiple cellular functions, including the cell adhesion, cell migration, cell cycle and DNA replication processes. Further, the in vitro experiment demonstrated that RSRC2 could inhibit the MDA-MB-231 cells and MDA-MB-453 cells clonogenic ability, adhesion, migration and invasion ability. The transcriptome array data analysis identified differentially expressed genes in the MDA-MB-231 shRSRC2 cells and control cells and identified SCIN, IL6, CXCL8, CXCL1, PTGS2, IL1B, NOS3, ICAM1, moesin and DCAF6, which were reported to be involved in cell proliferation, migration and cell adhesion [15,21,22,23,24,25,26,27,28]. The intersection set analysis of the bioinformatics database and transcriptome array data identified six common genes, FSTL1, GDF15, IQSEC2, KDM3A, LTC4S and TCTEX1D4, in which most of them were involved in tumor cell proliferation and growth [29,30,31,32,33,34]. These results demonstrate that RSRC2-correlated genes are involved in cell proliferation, cell adhesion, cell migration and invasion of TNBC.

Further, the ChIP-seq data identified that the DNA region bound to the RSRC2 protein could be at the promoter-TSS, intergenic, intron, 3′ UTR and exon regions. The Homer ChIP-seq analysis identified the motifs that could be bound by RSRC2 and, at the same time, could be bound by transcription factors CENPB, IRF4, ZNF354C, GRHL2, RARa, SOX5, SMAD4 PBX1, ZNF528 and EWSR1. Therefore, RSRC2 may play the role of a transcription factor in TNBC.

By integrating the Homer ChIP-Seq data and HTA 2.0 microarray data, we further explored the possible downstream target of RSRC2 and found SCIN had the motif occupied by RSRC2. Meanwhile, the STRING analysis suggested that SCIN may play a relatively independent role in the signal pathway regulated by RSRC2. SCIN expression was significantly decreased or increased in the RSRC2 overexpression or RSRC2 knockdown cells, suggesting that SCIN might be the downstream target of RSRC2. However, the Cistrome DB analysis showed that GRHL2 has the greatest potential for SCIN binding. Our previous study [35] demonstrated that GRHL2 was more highly expressed in breast cancer tissues than in normal tissues, and the high expression of GRHL2 was associated with a worse prognosis for breast cancer patients. Although the ChIP qRT-PCR testified GRHL2 could directly bind to the promoter region of SCIN, the GRHL2 expression did not increase or decrease following RSRC2 upregulation or downregulation, suggesting RSRC2 has no regulatory effect on GRHL2. Meanwhile, the luciferase reporter assay showed that RSRC2 negatively regulated the transcriptional activity of SCIN. The ChIP-qPCR results verified the binding of RSRC2 to the promoter of the SCIN gene. These results reveal the transcriptional regulation of SCIN by RSRC2 in TNBC. We found here that RSRC2 downregulated SCIN expression directly, not via regulating GRHL2.

The in vitro assays showed that downregulation or upregulation of RSRC2 promoted or inhibited MDA-MB-231 cells proliferation, adhesion, migration and invasion. The in vivo metastatic mouse model showed that RSRC2 knockout induced lung metastasis in the MDA-MB-231 cells. Meanwhile, the SCIN expression increased or decreased following RSRC2 downregulation or upregulation. The effect of SCIN on cancers was controversial among different investigators. There is a study [15] that demonstrates that SCIN knockdown inhibits breast cancer cell proliferation and induces apoptosis. However, the functional contribution of SCIN expression in TNBC progression has been relatively under-reported. In our present work, it is interesting to note that SCIN upregulation in the RSRC2-overexpressing cells or downregulation in the shRSRC2 or sgRSRC2 cells, respectively, reverses the cell adhesive ability, clonality and cell migration and invasion ability caused by RSRC2 upregulation or downregulation.

In summary, we demonstrated that RSRC2 served as a tumor suppressor in TNBC through negatively regulating SCIN-mediated cell function. Downregulation of RSRC2 was observed in breast cancer tissues through a large-sample bioinformatics multiple database analysis, especially in TNBC. The RSRC2 expression loss contributed to the accelerated proliferation, adhesion, migration and invasion of TNBC cells. The molecular experiments showed that RSRC2 transcriptionally repressed the promoter region of SCIN. The SCIN rescue experiment in RSRC2 downregulation or upregulation reversed the MDA-MB-231 cells function. Our study identifies SCIN as a novel transcriptional target of RSRC2 in TNBC cells and highlights the role of RSRC2 in TNBC by regulating the SCIN expression. Therefore, our study provided an innovative target for TNBC individualized treatment, and more RSRC2/SCIN-related signaling pathways should be identified in further studies.

## Data Availability

The data are contained within the manuscript.

## Supplementary Materials

The following supporting information can be downloaded at: https://www.mdpi.com/article/10.3390/cancers16010015/s1, Figure S1: Functional enrichment analysis showed that genes negatively related to *RSRC2* were mainly involved in regulation of cell migration, cell adhesion, focal adhesion, cell adhesion molecule binding and cell-substrate adherens junction. Figure S2: Functional enrichment analysis showed that genes positively related to RSRC2 were mainly involved in cell cycle and DNA replication process. Figure S3: RSRC2 expression affects clonality, migration and invasion of MDA-MB-453 cells possibly by regulating SCIN expression. Figure S4: Original western blots. Table S1: SCIN ChIP primer. Table S2: Correlation between RSRC2 mRNA levels and clinical data of breast cancer patients. Table S3: The mRNA expression profiles of MDA-MB-231 shRSRC2 cells and control cells by Human Transcriptome Array 2.0. Table S4: ChIP-seq data of MDA-MB-231 cells chromose DNA combined by RSRC2 protein.

## Abbreviations

RSRC2	arginine/serine-rich coiled coil 2
CHOL	cholangiocarcinoma
ChIP	chromatin immunoprecipitation
COAD	colon adenocarcinoma
ESCA	esophageal cancer
GBM	glioblastoma
HNSC	head and neck squamous cell carcinoma
Her2	human epidermal growth factor receptor 2
HTA	Human Transcriptome Array
KICH	kidney chromophobe cell carcinoma
KIRC	kidney renal clear-cell carcinoma
LIHC	liver hepatocellular carcinoma
LUAD	lung adenocarcinoma
LUSC	lung squamous cell carcinoma
SCIN	scinderin
STAD	stomach adenocarcinoma
THCA	thyroid carcinoma
TNBC	triple-negative breast cancer
UCEC	uterus endometrial carcinoma
