# Supplementary material for: RSRC2 Expression Inhibits Malignant Progression of Triple-Negative Breast Cancer by Transcriptionally Regulating SCIN Expression
Source: Cancers (Basel). 2023 Dec 19;16(1):15. doi: 10.3390/cancers16010015 (PMC10778392; doi:10.3390/cancers16010015)
Supplement: Supplementary file 1 [file cancers-16-00015-s001.zip › Supplementary Figures.pdf]

Supplementary Figures

Figure S1

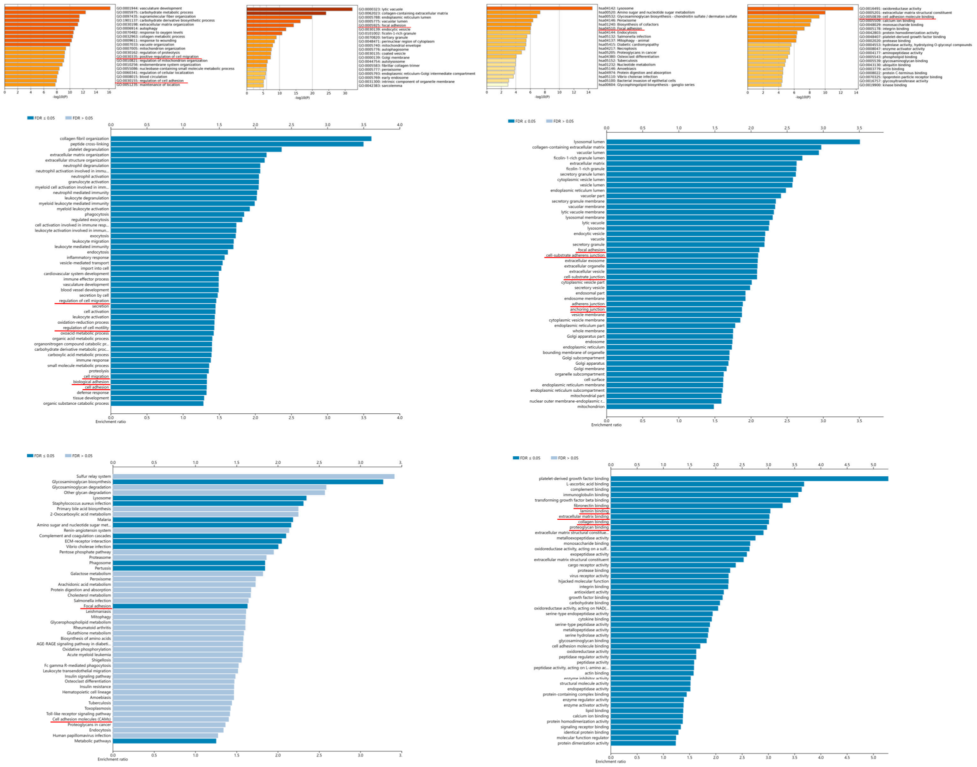

Figure S1 Functional enrichment analysis showed that genes negatively related to *RSRC2* were mainly involved in regulation of cell migration, cell adhesion, focal adhesion, cell adhesion molecule binding and cell-substrate adherens junction.

Figure S2

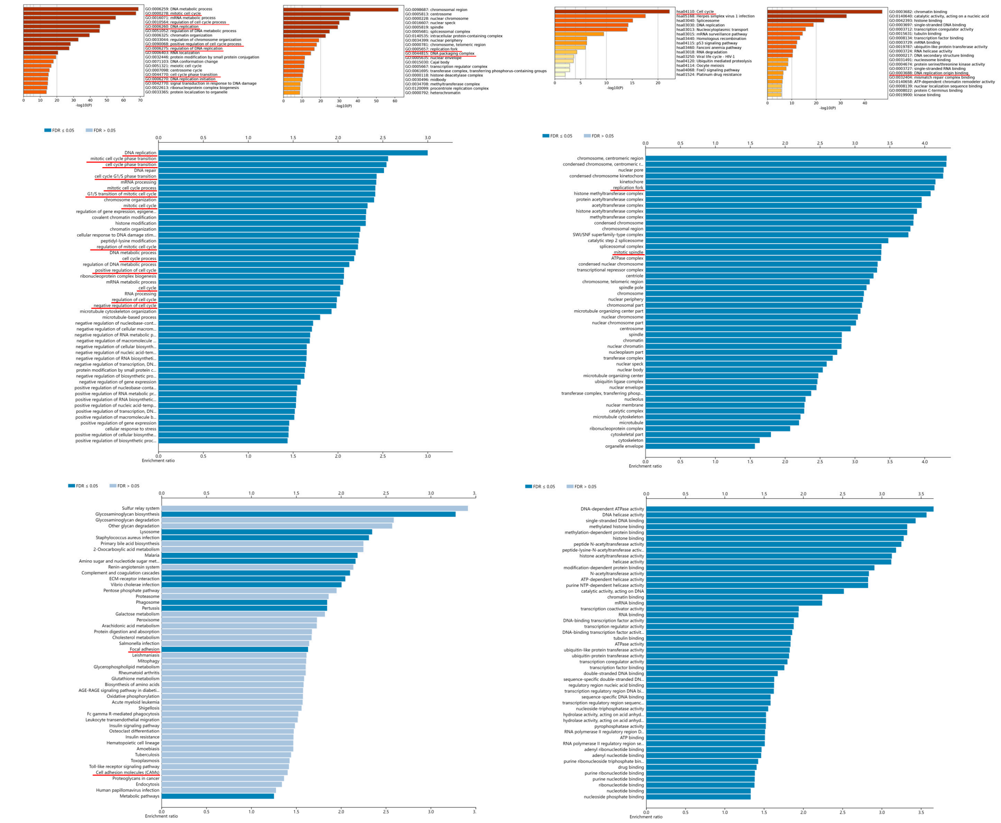

Figure S2 Functional enrichment analysis showed that genes positively related to *RSRC2* were mainly involved in cell cycle and DNA replication process.

Figure S3

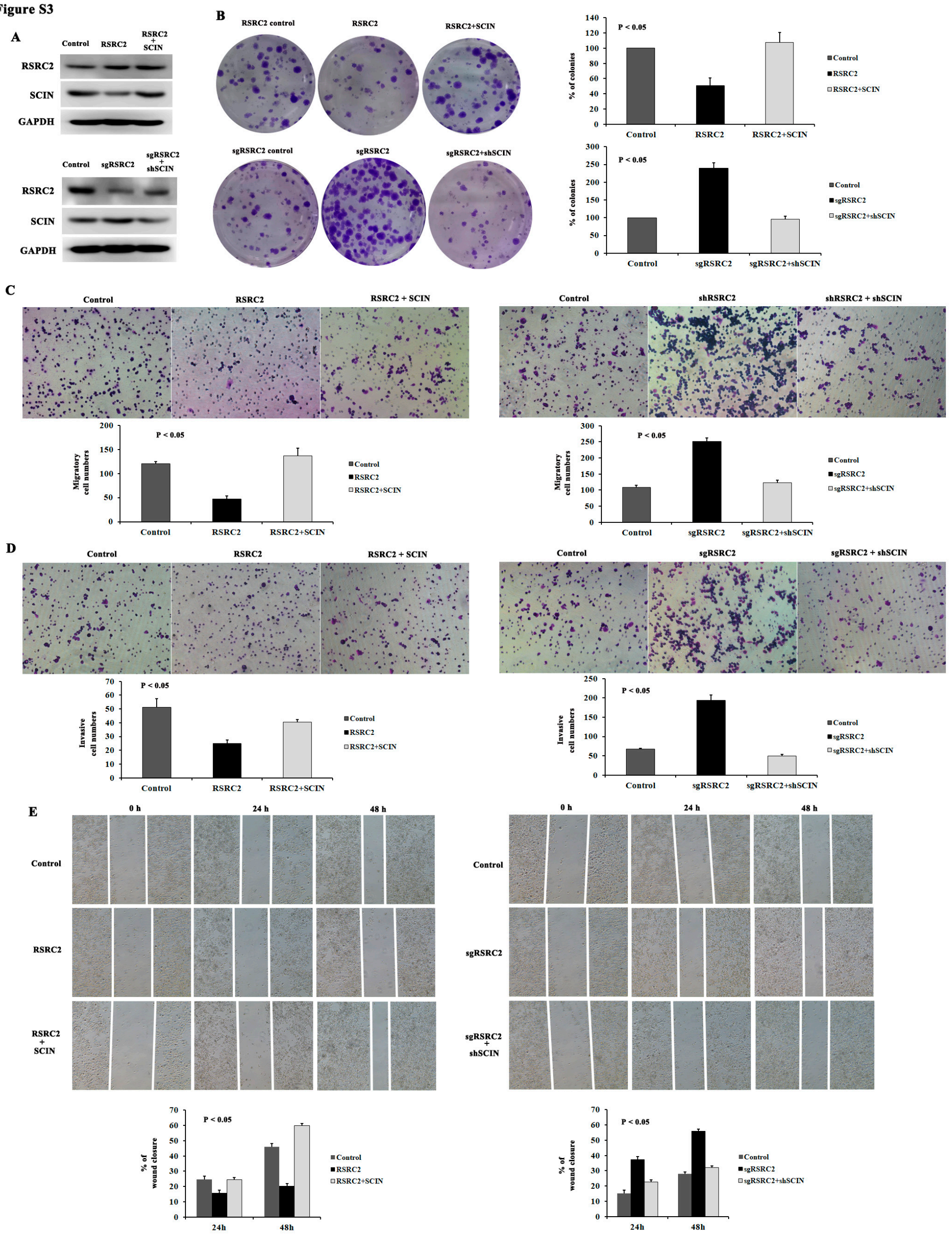

Figure S3 RSRC2 expression affects clonality, migration and invasion of MDA-MB-453 cells possibly by regulating SCIN expression. (A) The protein expression of RSRC2 in MDA-MB-453 cells overexpressing RSRC2 was significantly higher than that in the control cells, and the protein expression of RSRC2 in sgRSRC2 MDA-MB-453 cells was significantly lower than that in the empty vector transfected control cells. SCIN re-expression in RSRC2 overexpression cells and SCIN expression inhibition in sgRSRC2 cells by rescue experiments. (B-E) Rescue experiments indicated that SCIN upregulation in the RSRC2

overexpressing cells or downregulation in sgRSRC2 cells reversed the impacts on clonality (B), migration (C, E) and invasion (D) of MDA-MB-453 cells caused by RSRC2 overexpression or RSRC2 knockout. Error bars represent SD. The graphs represented three repeated experiments. ANOVA was used to compare multiple groups' means. $P < 0.05$ was considered to be significant.
