# Supplementary material for: RSRC2 Expression Inhibits Malignant Progression of Triple-Negative Breast Cancer by Transcriptionally Regulating SCIN Expression
Source: Cancers (Basel). 2023 Dec 19;16(1):15. doi: 10.3390/cancers16010015 (PMC10778392; doi:10.3390/cancers16010015)
Supplement: Supplementary file 1 [file cancers-16-00015-s001.zip › Table S2.pdf]

Table S2 Correlation between *RSRC2* mRNA levels and clinical data of breast cancer patients

Characteristic	Low expression of RSRC2	High expression of RSRC2	p
n	541	542	
T stage, n (%)			0.823
T1	133 (12.3%)	144 (13.3%)	
T2	321 (29.7%)	308 (28.5%)	
T3	67 (6.2%)	72 (6.7%)	
T4	18 (1.7%)	17 (1.6%)	
N stage, n (%)			0.177
N0	272 (25.6%)	242 (22.7%)	
N1	175 (16.4%)	183 (17.2%)	
N2	51 (4.8%)	65 (6.1%)	
N3	33 (3.1%)	43 (4%)	
M stage, n (%)			0.510
M0	451 (48.9%)	451 (48.9%)	
M1	12 (1.3%)	8 (0.9%)	
Pathologic stage, n (%)			0.085
Stage I	88 (8.3%)	93 (8.8%)	
Stage II	326 (30.8%)	293 (27.6%)	
Stage III	108 (10.2%)	134 (12.6%)	
Stage IV	12 (1.1%)	6 (0.6%)	
Age, n (%)			0.286
<=60	291 (26.9%)	310 (28.6%)	
>60	250 (23.1%)	232 (21.4%)	

Characteristic	Low expression of RSRC2	High expression of RSRC2	p
Histological type, n (%)			0.147
Infiltrating Ductal Carcinoma	389 (39.8%)	383 (39.2%)	
Infiltrating Lobular Carcinoma	91 (9.3%)	114 (11.7%)	
PR status, n (%)			< 0.001
Negative	221 (21.4%)	121 (11.7%)	
Indeterminate	3 (0.3%)	1 (0.1%)	
Positive	297 (28.7%)	391 (37.8%)	
ER status, n (%)			< 0.001
Negative	162 (15.7%)	78 (7.5%)	
Indeterminate	0 (0%)	2 (0.2%)	
Positive	359 (34.7%)	434 (41.9%)	
HER2 status, n (%)			0.236
Negative	269 (37%)	289 (39.8%)	
Indeterminate	7 (1%)	5 (0.7%)	
Positive	87 (12%)	70 (9.6%)	
PAM50, n (%)			< 0.001
Normal	28 (2.6%)	12 (1.1%)	
LumA	249 (23%)	313 (28.9%)	
LumB	75 (6.9%)	129 (11.9%)	
Her2	54 (5%)	28 (2.6%)	
Basal	135 (12.5%)	60 (5.5%)	
Age, median (IQR)	59 (49, 68)	58 (48, 67)	0.350
